# Gold Nanopyramid Arrays for Non-Invasive Surface-Enhanced Raman Spectroscopy-Based Gastric Cancer Detection via sEVs

**DOI:** 10.1021/acsanm.2c01986

**Published:** 2022-08-25

**Authors:** Zirui Liu, Tieyi Li, Zeyu Wang, Jun Liu, Shan Huang, Byoung Hoon Min, Ji Young An, Kyoung Mee Kim, Sung Kim, Yiqing Chen, Huinan Liu, Yong Kim, David T.W. Wong, Tony Jun Huang, Ya-Hong Xie

**Affiliations:** † Department of Materials Science and Engineering, University of California Los Angeles, Los Angeles, California 90095, United States; ‡ Department of Mechanical Engineering and Material Science, Duke University, Durham, North Carolina 27708, United States; § UCLA School of Dentistry, 10833 Le Conte Ave. Box 951668, Los Angeles, California 90095-1668, United States; ∥ Department of Medicine, Sungkyunkwan University School of Medicine, Samsung Medical Center, Seoul 135-710, Korea; ⊥ Department of Pathology and Translational Genomics, Sungkyunkwan University School of Medicine, Samsung Medical Center, Seoul 135-710, Korea; # Department of Surgery, Sungkyunkwan University School of Medicine, Samsung Medical Center, Seoul 135-710, Korea; ¶ Department of Bioengineering, University of California, Riverside, Riverside, California 92521, United States

**Keywords:** surface-enhanced Raman spectroscopy (SERS), small extracellular vesicle, machine learning, liquid biopsy, non-invasive cancer detection, gastric cancer

## Abstract

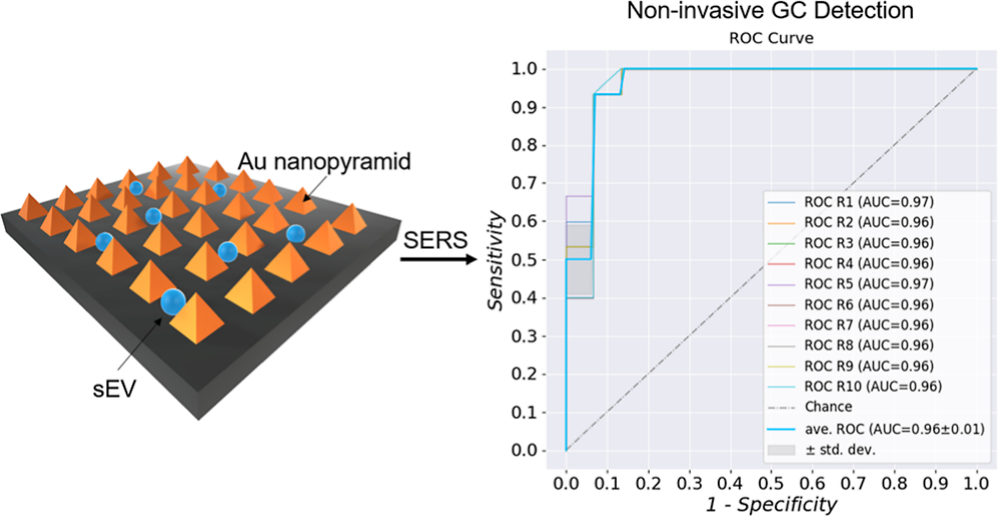

Gastric cancer (GC) is one of the most common and lethal types of cancer affecting over one million people, leading to 768,793 deaths globally in 2020 alone. The key for improving the survival rate lies in reliable screening and early diagnosis. Existing techniques including barium-meal gastric photofluorography and upper endoscopy can be costly and time-consuming and are thus impractical for population screening. We look instead for small extracellular vesicles (sEVs, currently also referred as exosomes) sized ⌀ 30–150 nm as a candidate. sEVs have attracted a significantly higher level of attention during the past decade or two because of their potentials in disease diagnoses and therapeutics. Here, we report that the composition information of the collective Raman-active bonds inside sEVs of human donors obtained by surface-enhanced Raman spectroscopy (SERS) holds the potential for non-invasive GC detection. SERS was triggered by the substrate of gold nanopyramid arrays we developed previously. A machine learning-based spectral feature analysis algorithm was developed for objectively distinguishing the cancer-derived sEVs from those of the non-cancer sub-population. sEVs from the tissue, blood, and saliva of GC patients and non-GC participants were collected (*n* = 15 each) and analyzed. The algorithm prediction accuracies were reportedly 90, 85, and 72%. “Leave-a-pair-of-samples out” validation was further performed to test the clinical potential. The area under the curve of each receiver operating characteristic curve was 0.96, 0.91, and 0.65 in tissue, blood, and saliva, respectively. In addition, by comparing the SERS fingerprints of individual vesicles, we provided a possible way of tracing the biogenesis pathways of patient-specific sEVs from tissue to blood to saliva. The methodology involved in this study is expected to be amenable for non-invasive detection of diseases other than GC.

## Introduction

Gastric cancer (GC) is the fifth most popular type of malignant tumor and the fourth most deadly worldwide with over one million new cases, leading to 768,793 deaths in 2020.^1^ Although the occurrence and mortality of GC have been on the decline, the five-year survival rate continues to be low.^2^ However, for patients diagnosed with GC at early stages, five-year survival rates of 95% or higher have been observed,^3,4^ demonstrating the overwhelming importance of early diagnosis and population screening. Early GC diagnosis requires reliable, cheap, and easy-to-operate screening methods that are yet to be available.^5^ Currently used screening methods such as barium-meal gastric photofluorography and upper endoscopy followed by biopsy can be costly and time-consuming.^6,7^ These procedures have also been shown to be associated with false negative rates, risks related with the rather invasive procedures, and other side effects.^8−10^ Recently, extracellular vesicles, especially sEVs, have become potential sources of biomarkers for cancer detection with easy access and minimal invasiveness.^11−14^

sEVs play crucial roles in cell-to-cell communications via the encapsulated cargos, which also reflect their parental cells.^15−18^ The stable existences in bodily fluids grant the potentials for them to be biomarkers for cancer liquid biopsy.^19−21^ By detecting these sEVs, opportunities exist for non-invasive cancer detection.^22,23^ For cancer patients, there co-exist sEVs from both normal and cancerous cells, each with their own characteristic biochemical cargo contents, forming different subtypes.^24,25^ One key challenge faced is that the vesicular liquid biopsy is a technique capable of examining individual sEVs, thereby distinguishing the sub-populations that belong uniquely to abnormal cells^26−28^

Techniques characterizing the physical properties of the sEVs include nanoparticle tracking analysis (NTA), dynamic light scattering,^29−31^ transmission electron microscopy (TEM), and scanning electron microscopy (SEM).^32^ Additionally, methodologies are employed for sEV content analysis.

Flow cytometry detects surface proteins on individual sEVs. Specific labels are normally required for desired specificities.^33−35^ Conventionally, it has a resolution around 100–300 nm.^36^ Despite the great efforts made toward lowering the detection threshold, requiring the known targets for labeling limits the capability of revealing the comprehensive biochemical information from sEVs.^37−39^

Western blot holds the gold standard for analyzing proteins in biological samples.^40^ It is a bulk detection method, which requires over 10^6^ sEVs during the process, thus losing the distinctive features from individual sEVs.^41^ Polymerase chain reaction (PCR) is widely used for genomic analysis of sEVs. DNA primers or templates are employed to initiate the process. sEVs normally need to be lysed before PCR.^42,43^ Recently, single-vesicle sequencing with the assistance of the DNA barcodes for detecting surface markers has been published that does not require the lysis of extracellular vesicles.^44^

The technologies mentioned are promising for analyzing sEVs from specific aspects with limitations. Among the alternative technologies, Raman spectroscopy has the potential to fulfill some of the unmet needs.

Raman spectroscopy provides structural “fingerprints” of different molecules and is thus capable of detecting and identifying biomedical substances by extracting the vibrational information of molecules.^45,46^ Raman spectra are correlated directly to the allowed phonon bands and are molecule-specific. However, the probability of Raman scattering events is extremely low (roughly 10^–6^).^47^ To boost the Raman spectral signal, metallic nanostructures can trigger surface-enhanced Raman spectroscopy (SERS).^48^ The spots on the surface where the electromagnetic field is intensified are referred to as “hotspots”, and the effective range of one hotspot is typically ∼200 nm.^49^ Two of the current major strategies of SERS for detecting diseases are as follows: first, SERS tagging for specific biomarker detection, and second, comparing spectral feature differences between the diseased and the control group, showing potentials in specific cases.^50−54^ Previously, we designed a SERS platform based on gold nanopyramids with single-molecule sensitivity.^55^ Furthermore, as a proof-of-concept, we applied this platform for testing sEVs, showing that sEVs from different cells of origin could be distinguished.^56^ For further interpreting SERS spectra with an objective spectral feature distinguishability, a data analysis mechanism is required. Machine learning has shown tremendous promise in meeting this challenge.

Machine learning has been introduced to assist diagnosis and analysis of big data in biomedical practices. The developed algorithm will first “learn” from the samples with known diagnoses via procedures to form a model/classifier.^57^ Such a model/classifier would then be used for diagnosing new patients.^58^ Despite the promising potential, one of the major hurdles faced during the “learning” process is mislabeling.^59,60^ Given the co-existence of sEVs from both normal cells and cancerous cells inside patients’ bodily fluids, the accuracy of machine learning-based sEV diagnostics will be negatively affected if all the vesicles from patients are labeled as the “patient group”.

Here, we applied our SERS gold nanopyramid platform to detect sEVs for non-invasive GC detection. Instead of using disease-specific SERS tags or focusing on particular spectral feature comparisons, the biochemical compositions of the collective Raman-active bonds were extracted from individual vesicles directly in the form of SERS spectra. We aimed to examine if these SERS spectra can be used as the sEV “fingerprints” for GC detection. For machine learning, we customized an algorithm to help correct the mislabeling issue in the clinical samples by sub-fractioning the measured sEVs. Leveraging the capabilities from both single-molecule SERS and machine learning, we proposed the technique as “SERS identification of molecules or SIM”. The results from SIM analysis of cell line-derived sEVs illustrated the existences of sEVs that were common to both the GC and the normal stomach tissues in addition to the characteristic ones. For clinical samples, vesicles were isolated from the tissue, blood, and saliva of donors from the GC patient group and the non-GC control group using an acoustofluidic platform (AFS) developed previously at the Duke University laboratory.^61^ The unique capabilities of the AFS including high efficiency and low processing time enable a better vesicle recovery rate and quality.^61^ The accuracies in identifying GC versus control were 90, 85, and 72% in tissue, blood, and saliva, respectively. “Leave-a-pair-of-samples out” analysis was performed to mimic the potential clinic applications of the platform. The result showed receiver operating characteristics (ROCs) with the area under the curves (AUCs) being 0.96, 0.91, and 0.65 in tissue, blood, and saliva cases, respectively. Additionally, nine patients’ unique sEV types were found to be existing across all three sample environments, opening a possibility for tracing the biogenesis of the GC patient-specific sEVs. The methodology involved in this study is amenable for non-invasive detection of diseases other than GC with further validation.

## Materials and Methods

### Cell Cultures

Three cell-lines, AGS (ATCC, CRL-1739), NCI-N87 (ATCC, CRL-5822), Hs 738.St/Int (ATCC, CRL-7869), were used in this study. The detailed protocol and the materials used in this study for the cell cultures is included in the supplementary Supporting Information.

### Tissue, Plasma, and Saliva Samples

For each donor, tissue, plasma, and saliva samples were collected respectively at the Samsung Medical Center in Korea. Tissue samples were collected from surgical resection during operation (GC patients) or tissue biopsy during endoscopic examination (non-GC control individuals). The collected tissues were stored at −80 °C until use. Plasma samples were collected using EDTA (ethylenediaminetetraacetic acid) tubes following the conventional clinical practice and stored at −80 °C until use.^62,63^ Unstimulated whole saliva collection was performed as described previously.^64^ From each subject, 5 mL of whole saliva was collected and centrifuged at 2,600*g* for 15 min at 4 °C. The SUPERase-In RNase inhibitor was added to the supernatant at 20 U/mL to stabilize salivary exRNA. The cell-free saliva supernatants were stored at −80 °C until use.

### SERS Substrate Fabrication

The recipe of the SERS platform used in this study was developed previously with the schematic and characterization results being provided.^55^ Basically, a single layer of self-assembled polystyrene (PS) balls (⌀ 500 nm) was generated with Langmuir–Blodgett patterning. The layer was transferred to a 4″ (001) silicon wafer with a layer of 50 nm SiO_2_ deposited on top. After further deposition of 50 nm Cr, the PS balls were removed using chloroform. After the SiO_2_ was exposed using reactive-ion etching, the silicon was etched using KOH. Inverted nanopyramids with sidewalls at 57.5° angles were created because of different etching rates along the [001] and [111] directions of silicon. Next, a 200 nm film of gold was deposited on the pitted surface using electron beam deposition and bonded to a carrier wafer using epoxy before lifting off.

### Ultracentrifugation

Cell culture supernatants were first centrifuged at 300*g* at 4 °C for 10 min and then at 2000*g* at 4 °C for 15 min to remove contaminating cells and apoptotic bodies, respectively. The supernatants were then further centrifuged at 12,000*g* at 4 °C for 45 min to remove cell debris. The clear supernatant was then filtered using 0.22 μm-pore filters, followed by ultracentrifugation at 110,000*g* at 4 °C for 70 min. The resulting pellets were re-suspended in pre-chilled PBS and again ultra-centrifuged at 110,000*g* and 4 °C for 70 min. The final pellet of sEVs was re-suspended in 50–100 μL of PBS for NTA measurement.

### AFS sEV Isolations

The detailed protocol of sEV isolation through an AFS in this study has been described in the previous publications.^61,65^

### Nanoparticle Tracking Analysis

The sample and PBS (Thermo Fisher, USA) sheath flow were independently controlled using a syringe pump (neMESYS, CETONI GmbH, Germany). Powered by a variable DC power supply (TP1505D, Tekpower, USA), a Peltier cooling system (TEC1-12730, Hebei IT, China) was used for avoiding excessive heat generation from the SAW device during sEV separation. An upright microscope (BX51WI, Olympus, Japan) combined with a CCD camera (CoolSNAP HQ2, Photometrics, USA) was used for monitoring the separation process. The sEV separation SAW device was powered by a function generator (E4422B, Agilent, USA) and an amplifier (100A250A, Amplifier Research, USA). After separation, the collected samples were analyzed by a NTA (Nanosight LM10, Malvern, England) system for getting the size distribution data.

### TEM

TEM validation follows our previous procedure.^65^ 4% paraformaldehyde (Sigma-Aldrich, St. Louis, MO) was used for fixing the isolated samples. 100 μL droplets of the fixed sample were covered with a 300-mesh copper grid support film (Electron Microscopy Sciences, Hatfield, PA) for absorption. The grids were washed with distilled water and then stained using uranyl acetate solution (Electron Microscopy Sciences). The grids were washed with distilled water again and dried at room temperature. An electron microscope (FEI, Hillsboro, OR) was used for observation.

### SEM

SEM was used to characterize the SERS substrate. Imaging was performed using Nova 230 with an accelerating voltage of 10 kV. The detector used was in the “through the lens” mode to detect secondary electrons, and the images were magnified at ×50,000 to ×55,000.

### Raman Spectroscopy

Before the Raman test, 5 μL of each sEV sample solution was deposited on the SERS substrate and dried. Raman measurements were performed using a Reinshaw inVia Raman spectrometer at room temperature. The laser excitation wavelength was 785 nm. The power used was 5 mW. Before measuring sEVs, the system was calibrated using the 520 cm^–1^ peak of silicon. Rough mapping was first performed at a step width of 2 μm to scout for the sEV locations. The exposure time was 0.2 s to avoid sample overheating. After an sEV was spotted, fine mapping was performed at a step width of 0.1 μm to collect characteristic spectra from the sEV sample. Again, the exposure time was 0.2 s to avoid sample overheating.

### Machine Learning Analysis

Approximately 50 to 70 different sEVs were obtained for each sample to produce spectra, which have 1023 Raman shifts in the range from 553 to 1581 cm^–1^ (biological information-rich region). Preprocessing steps were applied to alleviate the spectral signature fluctuations caused by sample variations, SERS platform heterogeneity, and instrument fluctuation. Particularly, fluorescence background subtraction and noise reduction were performed by batch processing based on asymmetric least square fitting and Savitzky–Golay filtering, followed by min–max normalization that proportionally compresses the original intensity range to [0, 1]. No initial feature selections or dimension reduction was performed prior to classification. To reveal the spectral differences among the three cell line groups, linear discriminant analysis (LDA) was used to reduce the dimensionality for visualization. For machine learning model development, predictive model establishment by supervised learning or classification is the core for the proposed technology. It requires appropriate complexity of the classifier to prevent both underfitting and overfitting for the purpose of generalizing the characteristic signature effectively. We use the conventional but powerful algorithm support vector machine (SVM) for classification tasks. Unsupervised learning or clustering analysis is performed by hierarchical clustering analysis with customized distance metrics; it investigates the intrinsic similarities among the analyte SERS signatures and serves as an auxiliary to classification. Randomly, 80% spectra from each of the groups (patient vs control) were selected for the model training and the rest 20% spectra were left out for cross-validation. 20 rounds of cross-validation were performed, with each round running independently to avoid overfitting. The prediction accuracy is the ratio between the number of correct predictions and the total number of predictions, following the equation eq 1. All the analyses are realized with Python.
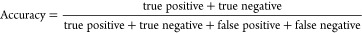
1

## Results and Discussion

### Experimental Process Flow

**Figure 1:**
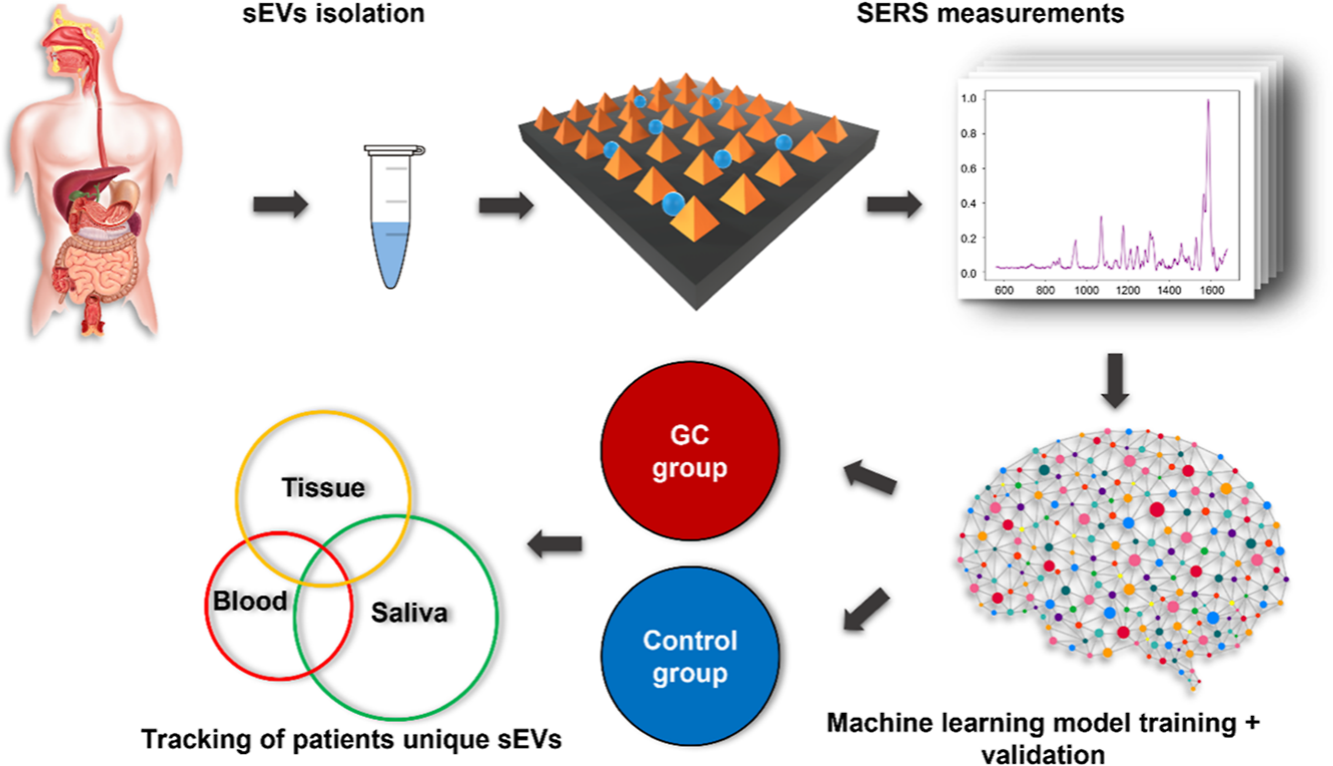
Schematic of SERS and machine learning for analyzing sEVs isolated from human samples.

The experimental process flow shown in Figure 1. Briefly, the experimental process can be described as three categories, sEV isolation followed by SERS spectra collections and data analysis. To begin with, sEVs were extracted from three types of samples (tissue, blood, and saliva) from human donors using an AFS. After isolation, the sEV analytes were dispersed on SERS substrates for measurements. Each sample droplet was 5 μL. After collecting SERS fingerprints, the machine learning algorithm (SVM) was employed to establish the distinguishability. Randomly selected 80% spectral data from each of the groups were used as the training set for building up the machine learning model, and the rest 20% spectra in each group were left out for testing the model’s predictability on cancer/control. The prediction results obtained from the testing phase were then compared with the true sample identification to calculate the accuracy. “Leave-a-pair-of-samples out” validation was performed to test the clinical applicability. Additionally, the SERS fingerprints from the patients’ unique sEVs were extracted from tissue, blood, and saliva followed by a cross-comparison for studying the possibility of tracing the vesicles through their SERS signatures.

### Overview of the sEV Samples and the Gold Nanopyramids

**Figure 2:**
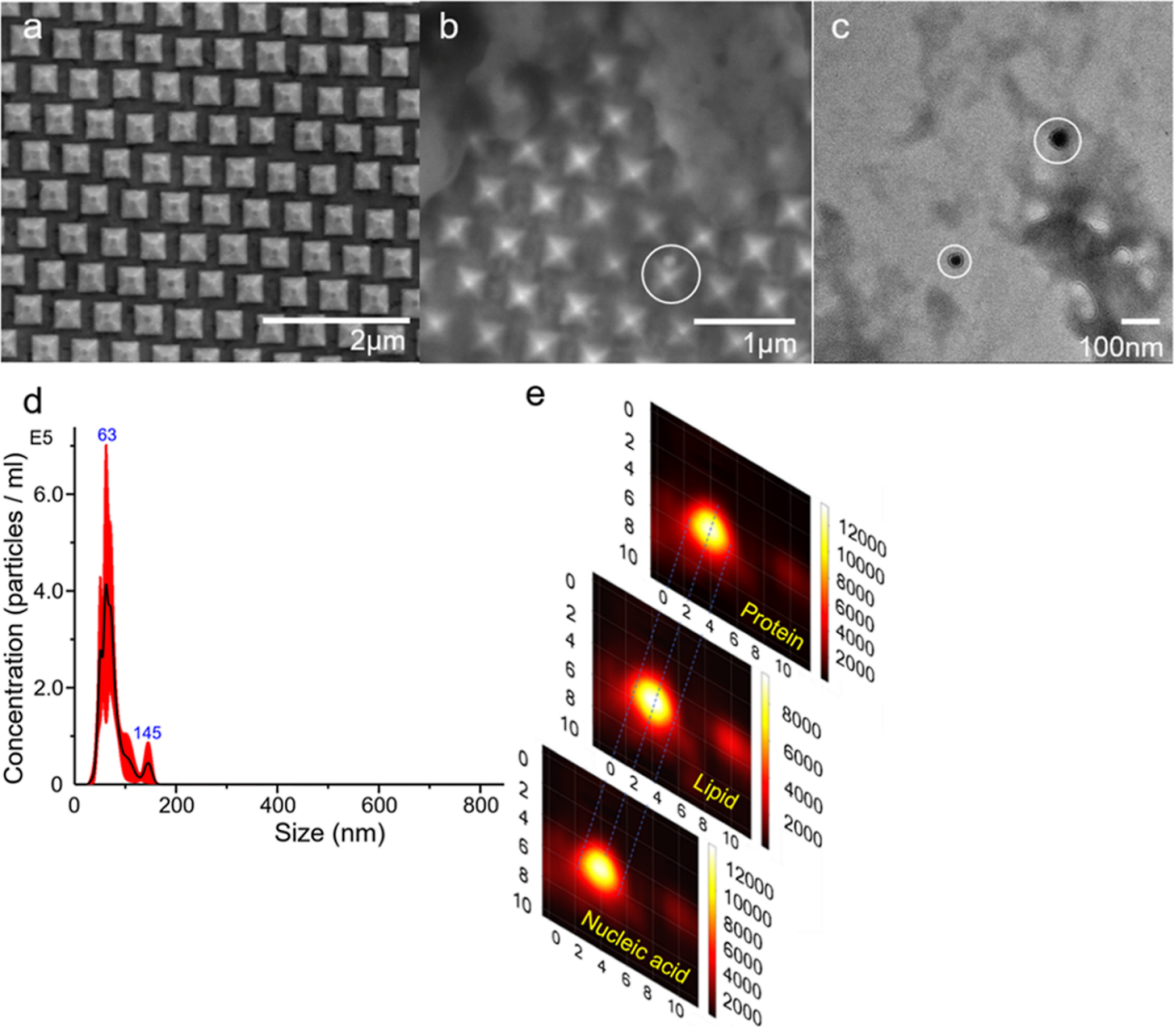
(a) SEM image of the SERS gold nanopyramids platform and (b) SEM image of the SERS substrate after sample solution introduction. (c) TEM image of isolated sEVs suspended in PBS, (d) NTA result of the isolated vesicles, and (e) SERS intensity maps generated with respect to nucleic acid, lipid, and protein from the same data spot.

Figure 2a shows the SEM image of the SERS gold nanopyramid substrate. The isolated sEV samples were characterized using NTA and TEM for verifications. Figure 2b shows the interaction between the sEV samples and the SERS substrate after the sample droplet had been introduced, suggesting intact vesicles lying in between the nano-pyramids. Figure 2c exhibits the TEM image of the sEV samples, where the sizes fall into the category of sEVs, and Figure 2d shows the NTA result of the size distribution of the vesicle samples. The substrate itself was Raman-inactive (SERS spectrum of the bare substrate is shown in Figure S1), and SERS spectra were collected from the areas with Raman spectral responses on a gold nano-pyramid array. Since the sEVs were ⌀ 30–150 nm in size, it was impossible to observe these vesicles directly under an optical microscope equipped on a conventional Raman spectroscopy system (shown in Figures S2 and S3). To verify, we performed SERS mapping with a super-fine grid on the said areas. The step width for each point in the mapping was set to be 100 nm, the minimal step width in a practical setup. Since the Raman laser is a Gaussian beam, a heat mapping based on the particular peak intensity fluctuations across a small area could be generated using such fine grid mapping. Three Raman intensity heat maps were plotted with respect to the Raman peak positions, representing phospholipids (1270 cm^–1^), nucleic acid (1341 cm^–1^), and protein (1123 cm^–1^). As shown in Figure 2e, the three heat maps were spherical, the shape of a vesicle, with the comparable sizes, suggesting the co-existing of the three substances essential to a vesicle. The three SERS intensity maps in Figure 2e show the raw shape of a single sEV. However, their sizes are larger than that of the actual vesicle. This is a result of convolution between a tightly focused laser beam of ∼1 μm diameter and a much smaller (∼100 nm) hotspot (the source of the Raman signal).^55,66^ The resulting diameter of the heatmap is limited by the larger of the two, in this case that of the excitation laser beam for Raman excitation of 1 μm. Nonetheless, the three SERS intensity maps show the shape of an sEV with comparable sizes consistently, suggesting the co-existence of the three substances, improving the rigor of the result. SERS mapping shows that the average spacing between sEVs is larger than 5 μm, much larger than the size of sEVs and the laser spot size. It needs to be pointed out that because of the instrumentational limitation, specifically the Raman laser spot size being larger than the sEV diameters, it is challenging to precisely map the morphology of an individual sEV. However, SERS signal intensity is quadratically dependent on the local electromagnetic field intensity, making the signal from one single plasmonic hotspot dominant.^67,68^ When utilizing our SERS platform, the hotspot size matches the size range of sEVs (∼100 nm).^55,56,66^ Such a feature allows the SERS signal from single sEVs to be collected one at a time. With the concentrations of the vesicles in study and the observation that the spectra indicating the existences of vesicles distributed randomly across the SERS mapping, the detection of sEVs in this study was single-vesicle-based from the statistical perspective. It should be noted that although the majority of the SERS spectra were derived from single sEVs statistically, there is a non-zero probability that occasional ones could be derived from more than one sEVs.

### SIM Analysis of sEVs from Cell Lines

**Figure 3:**
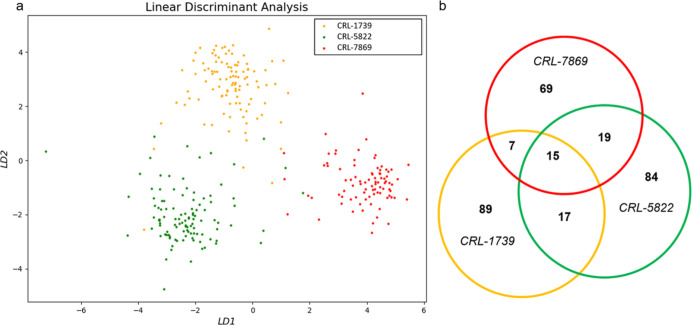
(a) LDA result distinguishing the SERS spectra of the sEVs derived from cell lines as three groups and (b) statistical results of SERS signature comparisons among individual vesicles.

Before working on the patient samples, we first used cell line-derived sEVs by ultracentrifugation to establish the capability of SIM to detect and analyze single sEVs. More importantly, analyzing sEVs from cell lines provides the information of single-sEV heterogeneities, given the purest forms of parental cells. Such knowledge laid the foundation and set the expectations for the further studies with clinical samples. Two GC cell lines (CRL-1739 and CRL-5822) and one normal stomach tissue cell line (CRL-7869) were involved. SEVs from each of the cell lines were isolated from the culture medium using ultracentrifugation before being dropped onto the gold nano-pyramid substrate. Within each sample, SERS measured sEVs one at a time, generating one spectrum per vesicle. For CRL-1739-, CRL-5822-, and CRL-7869- derived sEVs, 115, 106, and 86 vesicles were measured by SERS, respectively. After spectral collection, LDA was applied first to study the distinguishability of SIM in the three groups. Figure 3a showed that the SERS spectra from the three groups could be distinguished, with a variety of sEVs within each of the sample groups revealed by SIM. Inherently, LDA is a technique used to separate different groups of data. In order to directly and objectively compare the SERS fingerprints of individual sEVs for determining the common and characteristic vesicles among the three groups, a machine learning-based (SVM) SERS spectral feature comparison mechanism was introduced. Figure 3b exhibits the results of comparing the SERS fingerprints of individual vesicles across the three cell line groups. SERS spectra of the common vesicles could be found among different sample groups in the cell line-derived sEV groups, shown in Figures S4–S7. Internal spectral variations existed within each of the sEV types, but the spectral differences across different vesicle types outweighed the internal spectral variations as measured from the Euclidean distance between the nearest neighbors. The results of analyzing the three cell lines illustrated the existences of sEV populations common to both GC tissue and the normal stomach tissue released groups even in the cell line forms. Such observation further indicated the existences of sEV populations common to both GC/non-GC groups in the clinical bodily fluids. When analyzing single vesicles, it is necessary to sort out the common populations before an accurate detection could possibly be made.

### SIM Analysis of Clinical Samples for GC Detection

**Figure 4:**
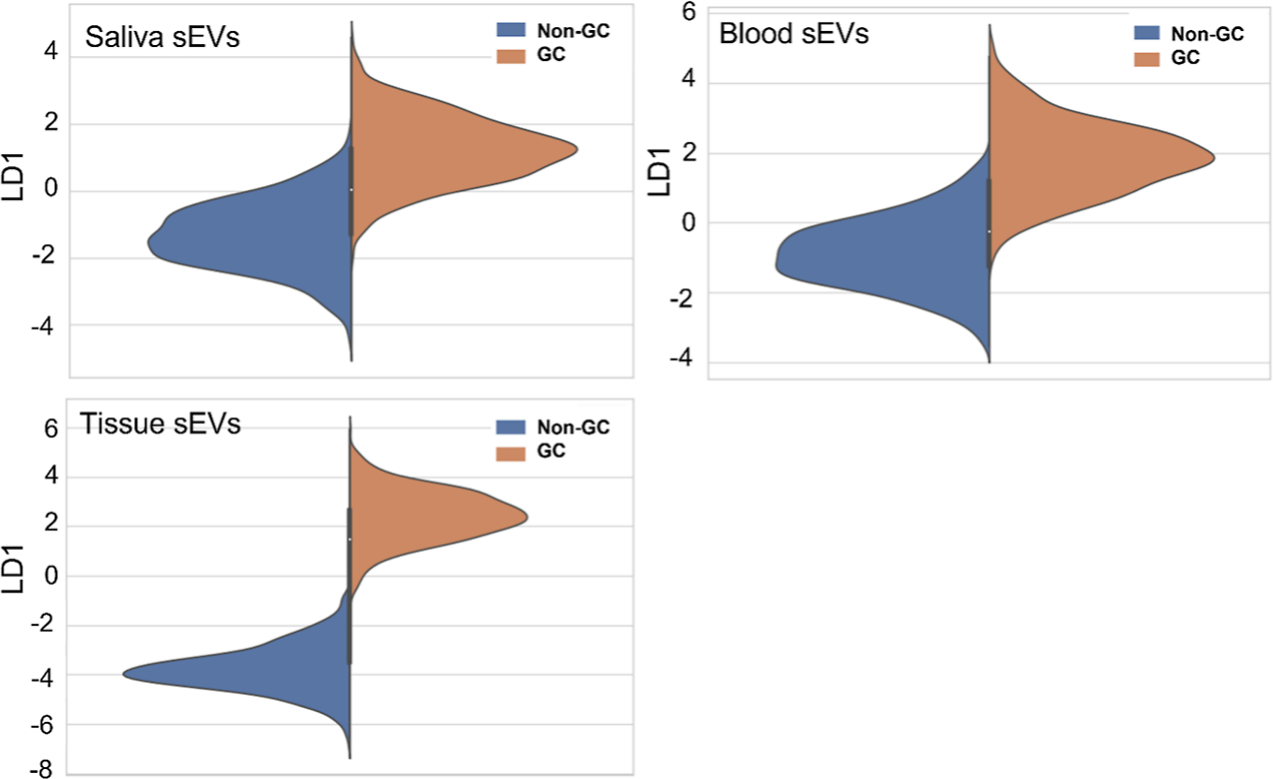
LDA results comparing the SERS spectra of sEVs in tissue, blood, and saliva.

Next, we applied the SIM platform to analyze the tissue-, blood-, and saliva- derived sEVs from GC patients and non-GC controls (*n* = 15 for each of the groups). To address the low-vesicle concentration issue, the AFS that was developed previously at the Duke University laboratory was employed for better sEV recoveries during the isolation from the human samples to avoid the well-known vesicle loss during the ultracentrifugation isolation.^65^ On average, 60 different data spots were measured by SERS for each of the sample droplets. Compared to cell lines as the sEV-extracting sources, we expected two key factors that could increase the complexities when studying vesicles from the human bodies. First, samples from human bodies, especially the bodily fluids, have lower vesicle concentrations compared to those of cell culture media. Second, the populations of the vesicles that are common to both patients and the control group are expected to be more in the bodily fluids because the normal cells inside a patient’s body secrete the same/similar types of vesicles as the ones inside a non-cancer control person, leading to the mislabeling problem in machine learning and further damaging the detection accuracy. The LDA results of the clinical samples are shown in Figure 4. The *y* axes are LD1 scores, and the Gaussian-like curves record the spectral distributions according to the LD1 scores. We observed that sEV samples in saliva and blood showed overlapping between the GC and the non-GC groups from LD1 equals −2 to 2. On the other hand, such overlapping was hardly observed in the tissue sEV samples. Such results indicated that some of the sEVs in saliva and blood shared similar/common SERS spectral features between GC and non-GC, further inferring the existences of normal sEVs inside patients’ bloods and saliva. Such observations verified our hypothesis, given that the biochemical compositions of the sEVs could reflect their parental cells. In addition, the LDA results suggested that the mislabeling issue was inevitable, thus requiring relabeling before the SVM classification model training, especially in blood and saliva samples.

**Figure 5:**
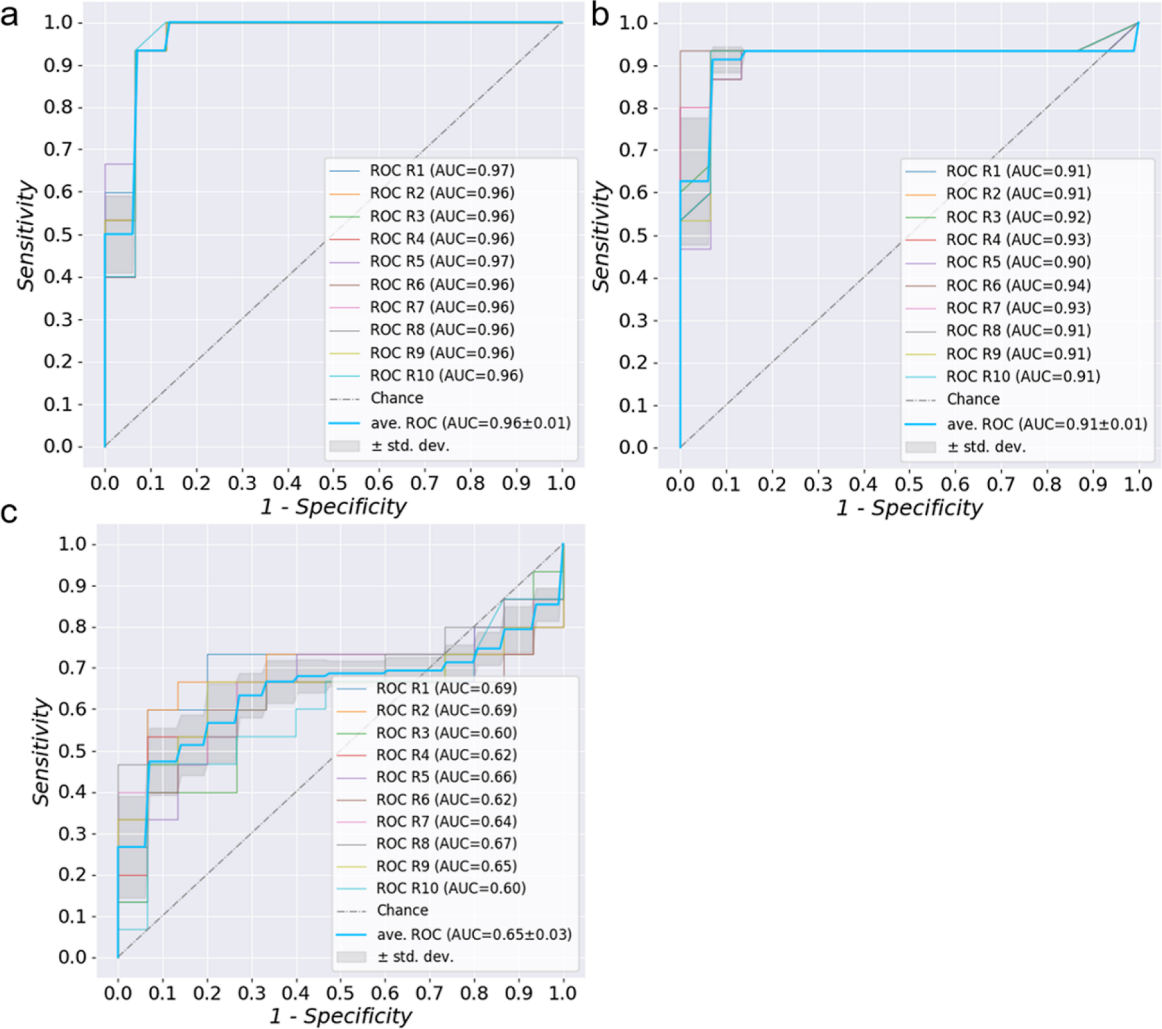
ROC curves of the “leave-a-pair-of-samples out” validation for tissue sEVs (a) blood sEVs (b) and saliva sEVs (c).

To address the mislabeling problem in machine learning, a relabeling process was involved before training through sub-fractioning of individual sEVs from patients based on their biochemical compositions reflected in the SERS fingerprints. As shown in Figure S8, we first compared the SERS fingerprints of individual sEVs between the patient and the control group, extracting the sEV types that uniquely existed in the patient group (PP). Such a process was carried out by cross-comparing the spectral features of the individual spectra between the GC and the non-GC group. The spectra in the GC group that shared common spectral features with the ones in the non-GC group were related to the normal sEVs inside the patients’ bodies, given that the sEVs could reflect their parental cells. They were further separated from the spectra found only in the GC group (PP). Then, only the SERS signatures from the PP group were labeled as “GC” and others were labeled as “control” for the machine learning model training. Such a process was referred as “relabeling” and helped correcting the mislabeling issue mentioned previously. Cross-validation of randomly selected 20% SERS spectra of sEVs from each of the two groups was carried out to study the detection accuracy. The selected 20% was intentionally left out during the training to avoid the information leak during the training, improving the rigor. For analyzing the SERS fingerprints collected from sEVs of tissue, blood, and saliva, both non-relabeling and relabeling methods were involved, and the detection accuracies are exhibited in Table 1. Each of the results was an average of 20 rounds of cross-validation (σ^2^ ≤ 0.000541). In general, tissue-derived sEVs held the highest accuracy of distinguishing, followed by the blood-derived and saliva-derived vesicles. With relabeling, the detection accuracies were improved for sEVs collected from blood and saliva but not significantly changed for the samples from tissue. For the machine learning model training and cross-validation, the training data set and the validation data set selected have no overlap. Such procedure avoids the pitfall of information leak, that is, the machine learning program has been given some clue about the identity of the samples used for cross-validation, leading to a falsely high accuracy of detection. The relabeling helps correcting the mislabeling issue due to the inevitable existence of normal vesicles in the samples of the cancer patients. To further explore the potential of our analysis in the clinical applications, we performed “leave-a-pair-of-samples out” validation using the relabeled spectra. In such validation, SERS spectra from a randomly selected GC patient and a randomly selected control individual were intentionally excluded from the training set and used as the test set. The random pairing and “leaving out” continued but did not include samples that had been selected before. One round ended when every sample had been “left -out” once. To test statistical fluctuations, we performed 10 rounds for each of the tissue, blood, and saliva samples. Figure 5 shows the resulting ROCs. The averaged AUCs were reported to be 0.96, 0.91, and 0.65 in tissue, blood, and saliva, respectively. It needs to be pointed out that “leave-a-pair-of-samples out” cannot completely substitute the actual clinical blind test. However, to our understanding, such validation offers a chance to test the platform’s clinical applicability by providing a scenario closer to the blind test than normal cross-validation. The result suggests that apart from tissue samples, blood holds a greater potential than saliva as a source for sEV-based GC liquid biopsy by involving the minimal invasiveness of puncture.

**Table 1:** SVM Model Prediction Accuracies in Cross-Validation

	tissue sEVs		blood sEVs		Saliva sEVs	
	non-relabel	relabel	non-relabel	relabel	non-relabel	relabel
accuracy (% of correct predictions)	90%	89%	72%	85%	58%	72%

Our GC/non-GC distinction results show that there existed improvements in detection accuracies with relabeling in saliva and blood samples but no significant change in the tissue samples. This observation could be explained as the reflection of the different relative population of the GC-specific sEVs. The sEV populations derived from the patients’ tissues contain the highest concentration of the patient-characteristic vesicles. Such concentration drops when these sEVs circulate in the bodily fluids, where they are joined by vesicles released by other (non-GC) organs. We acknowledge that such a single-sEV detection mechanism has its fundamental limitation on the throughput, which is inversely proportional to the required number of sEVs to be examined per patient sample in order to perform accurate diagnosis. Nonetheless, our study reveals the feasibility of non-invasive GC detection/screening by analyzing the composition information of the collective Raman-active bonds inside single sEVs isolated from blood and saliva using SIM. SERS measurements provided molecular fingerprints from single vesicles, and the machine learning algorithm offered the ability to distinguish among the various sub-fractions of the sEVs with objectivity and rigor. Despite the complexity of the microenvironments inside human bodies, the SIM platform has been shown to be capable of reaching the detection accuracy of 90, 85, and 72.0% with the reported AUCs of 0.96, 0.91, and 0.65 in the “leave-a-pair-of-samples out” validation in tissue, blood, and saliva, respectively. The difference in detection accuracy makes intuitive sense, considering the difference in the relative concentration of GC-specific sEVs in these three sources. Needless to say, blood and saliva are the more non-invasive sources of sEVs and thus have higher promise in clinical practice. Effort is in progress to further optimize the machine learning algorithm with the goal of improving the detection accuracy.

With the development of the field, promising studies related to the innovations and the applications of SERS substrates for cancer detection via extracellular vesicles have been published. Shin et al. (2020) reported a combination of SERS and deep learning for early-stage lung cancer detection via sEVs derived from blood with AUC >0.9.^51^ To achieve such detection, spectral features from the test set were classified based on the degrees of similarities to the training set.^51^ In our present study, we observed a comparable AUC in “leave-a-pair-of-samples out” validation with the blood sample, but such scores dropped with the saliva samples. From the clinical and practical perspectives, the observation of detection accuracy discrepancies in blood and saliva could serve as an indication for biofluid selections in the liquid biopsy-based GC detections. Dong et al. (2020) focused on one specific content and reported that the variations of the SERS signal intensity of protein phosphorylation inside sEVs between the control and patients could serve as the indicator for detecting prostate, liver, lung, and colon cancers.^69^ Carmicheal et al. (2019) utilized SERS gold nanoparticles and machine learning for pancreatic cancer detection.^70^ SEVs from serum were measured by SERS from 20 (*n* = 10 for from cancer/control) individuals, and machine learning algorithm prediction results indicated the diagnostic potential and the bio-variability dragging the effectiveness due to the diverse origins of the serum sEVs.^70^ Rojalin et al. (2020) reported a porous scaffold SERS platform that could prevent vesicles from drying during the SERS measurements with PCA, indicating clear separations of the SERS spectra of extracellular vesicles (sEV and larger ones) collected from two control individuals, two ovarian cancer patients, and two endometrial cancer patients.^71^ In addition, the dragging of detection accuracy by trypsinization of clinical vesicles was illustrated.^71^ Apart from analyzing the spectral features directly, the modified SERS substrate for vesicle immobilization allows quantitative analysis of the captured sEV populations between the cancer and the control group through the comparison of intensities of the SERS indicator as demonstrated by Banaei et al. (2021).^72^ In our present study, the single-vesicle-based analysis enables identification and discrimination among different subtypes of sEVs within the same sample group. The relabeling process for enhancing the detection accuracy cannot be carried out without the signatures from each of the individual vesicles. In addition, the identification of single vesicle offers the feasibility of tracing the trafficking pathways of the patients’ sEVs from the cancer tissues to the bodily fluids based on their SERS fingerprints. It opens a new possibility for the further understanding of the sEV biogenesis, specifically the pathway of sEV trafficking from the point of secretion to entering body fluids.

### Tracking sEVs Uniquely Belonging to the Patient Group

**Figure 6:**
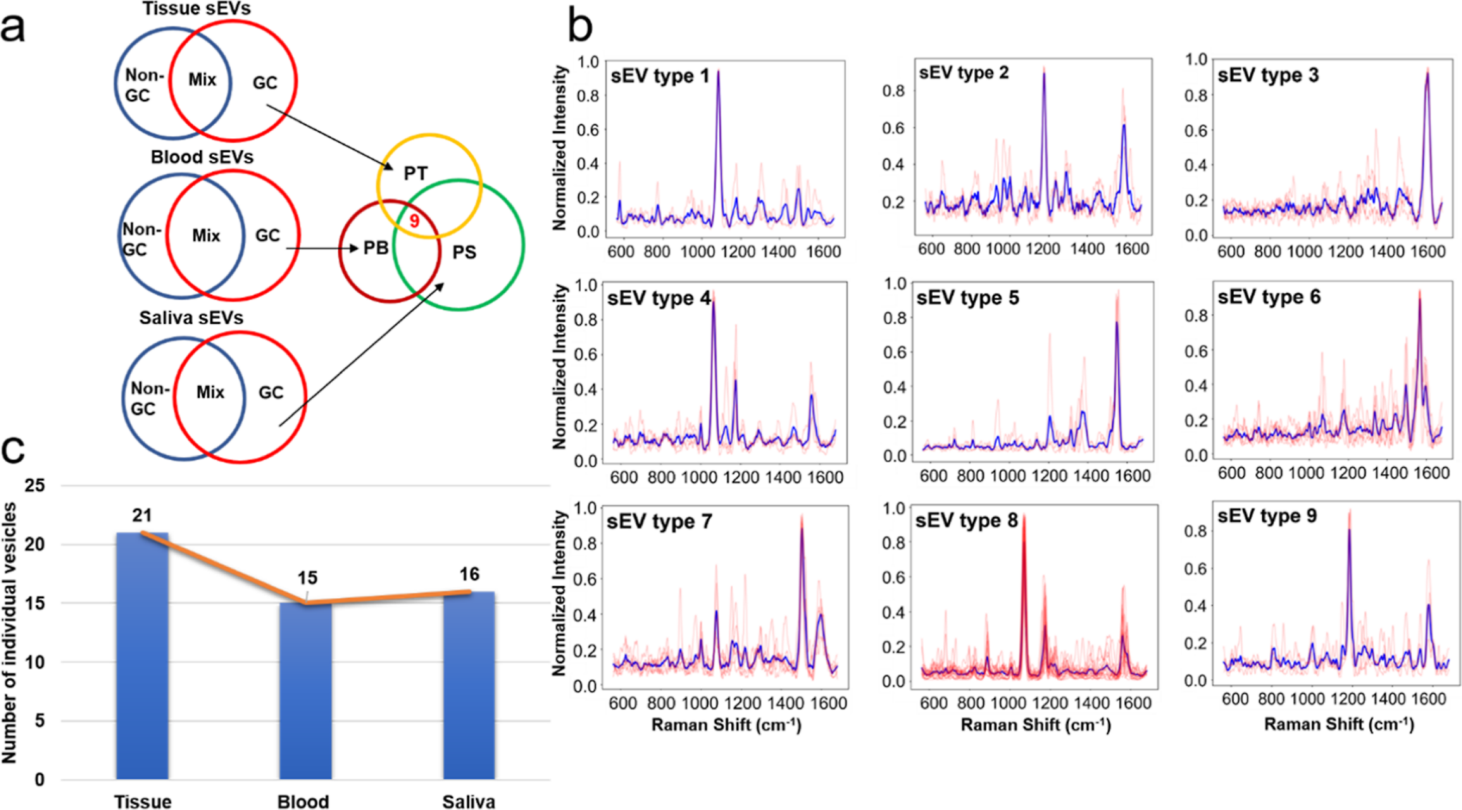
(a) Schematic of tracking patients’ unique sEVs and (b) superimposed SERS spectra (red) and the corresponding average spectrum (blue) of the patients’ unique sEVs existed across tissue, blood, and saliva. Horizontal-axis: Raman shift (ranging from 553 to 1581 cm^–1^). Vertical-axis: Normalized intensity 0–1; (c) distribution of all the individual sEVs of the nine types presented in (b).

After illustrating the GC detectability of SIM by analyzing the SERS signatures of single sEVs, we further looked at the vesicles that uniquely belonged to the patients (PP) in tissue, blood, and saliva. The goal was to study the feasibility of using SIM to track single sEVs inside the body based on their SERS fingerprints, given that the published studies have shown that different vesicle phenotypes exist in different parts of the human body.^73,74^ As shown in Figure 6a, after extracting the PP group from tissue, blood, and saliva, respectively, as mentioned (PT, PB, and PS), the SERS spectral comparison was introduced to identify the common vesicles existing across tissue, blood, and saliva. Nine sEV types were identified, existing across all three conditions. Figure 6b exhibits the superimposed SERS spectra together with the averaged spectrum for each of the sEV type identified, and Tables S1–S9 contain the tentative Raman peak assignments for the major peaks of the nine vesicle types. Within the nine sEV types, the result of studying the source of each individual vesicles is shown in Figure 6c, suggesting the population of patient unique sEVs dropping from tissue to blood/saliva, which is consistent with the current understanding about cancerous sEV circulation. The results of tracking sEVs uniquely belonging to the patient group through their SERS signatures opened a new possibility for tracing vesicle circulations from the tissue of origin to the bodily fluids. In addition, our tracking studies shed light on the impact of the sample of origin on the diagnostic accuracy and practicality: tissue is the most invasive source but contains the highest concentration of disease-specific sEVs, whereas saliva is the least invasive but contains less concentration of the disease-specific vesicles.

The identification of nine types of the patients’ unique sEVs existing across tissue, blood, and saliva illustrates the feasibility of using SIM for tracking patients’ vesicles. However, we did not observe that one or more of the nine identified sEV types existed across all patients. The nine types of sEVs not being shared among all cancer patients could possibly be due to the small sample (sEV) size, currently limited by our SIM throughput. Using the commercially available Raman spectrometer (InVia by Renishaw) designed for research instead of medical laboratories, the routinely achievable throughput is approximately 15–20 sEVs per hour. It should be pointed out that this throughput is by no means intrinsic to SERS analysis of biological samples. Several areas of the spectrometer could be automated to conceivably improve the Raman mapping throughput by orders of magnitude. With significantly increased sample size per patient/healthy control, the probability of mislabeling (identifying an sEV type to be cancer simply because such an sEV happened to be absent from a limited size of healthy control samples) will decrease significantly. Nonetheless, our study has shown a promising pathway for the development of an evidence-based procedure factoring in clinical considerations. With necessary clinical trials for validating, the methodology involved in this study is amenable for non-invasive detection of diseases other than GC and further understanding and tracing of the biogenesis pathway of the sEVs.

## Conclusions

In this study, a gold nanopyramid platform was applied for SERS measurements of sEVs, exploring the feasibility of non-invasive GC detection. It demonstrates the feasibility of non-invasive GC detection/screening by analyzing single sEVs isolated from blood and saliva by SIM, a combination of single vesicle SERS and machine learning. The data obtained from sEVs derived from tissues served as the references for the possible tracking of patient unique vesicles. The distinguishing accuracy of sEVs between GC patients and non-GC controls is 90, 85, and 72% with the AUC in the “leave-a-pair-of-samples out” validation to be 0.96, 0.91, 0.65 in tissue, blood, and saliva, respectively. Nine sEV types were identified, existing across all three conditions. The methodology developed in this study has the potential to be applied for the detection of other cancers using individual sEVs with further studies for verification.

## Supporting Information Available

Methods for cell culture, harvesting, and storage; method for machine learning analysis; SERS spectrum of the bare gold nanopyramid arrays substrate; optical images of the gold nanopyramid array substrate before and after sample introduction; SERS spectra of sEVs derived from cell lines common to two or more parental cells; LDA results of the clinical samples; and Raman peak assignments for the nine patients’ unique sEV types in tissue, blood, and saliva (PDF)
