# Supplementary material for: Gold Nanopyramid Arrays for Non-Invasive Surface-Enhanced Raman Spectroscopy-Based Gastric Cancer Detection via sEVs
Source: ACS Appl Nano Mater. 2022 Aug 25;5(9):12506–17. doi: 10.1021/acsanm.2c01986 (PMC9513748; doi:10.1021/acsanm.2c01986)
Supplement: Supplementary file 1 — an2c01986_si_001.pdf [file an2c01986_si_001.pdf]

Supporting Information:

Gold Nanopyramid Arrays for Non-Invasive Surface-Enhanced Raman Spectroscopy-Based Gastric Cancer Detection via sEVs

Zirui Liu[1], Tieyi Li[1], Zeyu Wang[2], Jun Liu[1], Shan Huang[1], Byoung Hoon Min[4], Ji Young An[4], Kyoung Mee Kim[5], Sung Kim[6], Yiqing Chen[7], Huinan Liu[7], Yong Kim,[3] David T.W. Wong[3], Tony Jun Huang[2], and Ya-Hong Xie[1]*

[1]Department of Materials Science and Engineering, University of California Los Angeles, Los Angeles, CA 90095, USA

[2]Department of Mechanical Engineering and Material Science, Duke University, Durham, NC 27708, USA

[3]UCLA School of Dentistry, 10833 Le Conte Ave. Box 951668, Los Angeles, CA 90095-1668, USA

[4]Department of Medicine, Samsung Medical Center, Sungkyunkwan University School of Medicine, Seoul 135-710, Korea

[5]Department of Pathology and Translational Genomics, Samsung Medical Center, Sungkyunkwan University School of Medicine, Seoul 135-710, Korea

[6]Department of Surgery, Samsung Medical Center, Sungkyunkwan University School of Medicine, Seoul 135-710, Korea

[7] Department of Bioengineering, University of California, Riverside, Riverside, CA 92521, USA

*To whom correspondence should be addressed:

Ya-Hong Xie

Department of Materials Science and Engineering,

University of California Los Angeles, Los Angeles, CA 90095

E-mail: yhx@ucla.edu

Phone number: (310) 259-6946

Cell culture, harvesting, and storage

Three cell lines (AGS, NCI-N87, Hs 738.St/Int) were used in this study. AGS (ATCC, CRL-1739) are epithelial cells from the adult female stomach with gastric adenocarcinoma. NCI--N87 [N87] (ATCC, CRL-5822) are epithelial cells from male gastric carcinoma. Hs 738.St/Int (ATCC, CRL-7869) are fibroblasts from 18 weeks gestation fetus. All cell lines were purchased from ATCC

Cell culture and medium collection:

In this study, all the fetal bovine serum (FBS; ATCC, 30-2020) were pre-treated to remove the sEVs. To prepare the complete culture medium for these three different cell lines, F-12K Medium (ATCC, 30-2004), RPMI-1640 Medium (ATCC, 30-2001), and Dulbecco's Modified Eagle's Medium (ATCC, 30-2002) were supplemented with 10% of pre-treated FBS and 1% of penicillin-streptomycin (P/S; ATCC, 30-2300) for AGS, NCI-N87, and Hs 738.St/Int, respectively. All the cell lines were thawed in a 37 °C water bath for 1 min and then transferred into a centrifuge tube containing 9.0 mL complete culture medium and spun at 125 x g in a centrifuge (Rotor F-35-6-30, 5430, Eppendorf, Germany) for 5 minutes to remove the dead cells. The cell pellets were resuspended with the complete culture medium and dispensed into a 75 cm^2 culture flask (T-75 flask; MSPP-90076, VWR, USA). Cells were cultured at 37 °C in a humidified incubator (MCO-19AIC, SANYO, USA) with 5% CO_2 and 95% air. Media were collected and changed every 2 - 3 days.

Cell harvesting and storage:

The cells were harvested when the flask reaches over 90% confluency and stored in liquid nitrogen vapor. The medium was aspirated from the cell culture flask and collected for the study. The flasks were rinsed with 10 mL Dulbecco's Phosphate Buffered Saline (D-PBS; ATCC, 30-2200), 2 -3 mL of Trypsin-EDTA solution (ATCC, 30-2101) were added to remove all traces of serum that contains trypsin inhibitor for 5 minutes. Then 6 - 8 mL of complete growth mediums were used to resuspend the cells, the cell suspension was transferred to a centrifuge tube and spun at 125 x g for 5 minutes. The cell pallets were collected and added into a cryovial (5000-1020, Thermo Fisher Scientific, USA) contains complete culture medium with 5% (v/v) Dimethylsulfoxide (DMSO; ATCC, 4-X) after aspirating out the supernatant medium. The cryovial was placed into a CoolCell (43200, Corning, USA) to freeze down to -80°C. After 2-3 days, store the cells in a liqiud nitrogen tank at vapor phase.

Figure S1. Spectrum of the bare SERS substrate

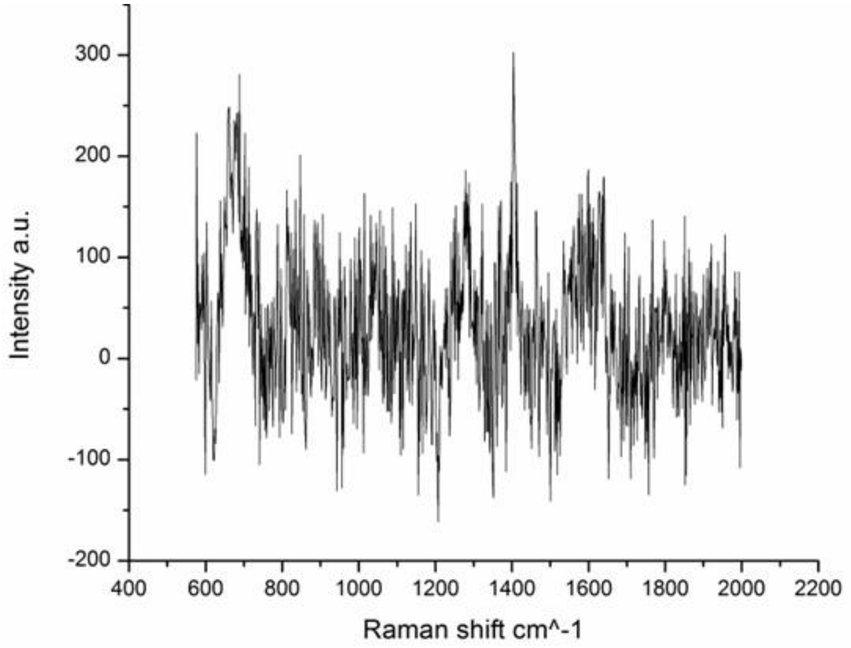

Figure S2. Optical microscope image of bare gold nanopyramids substrate

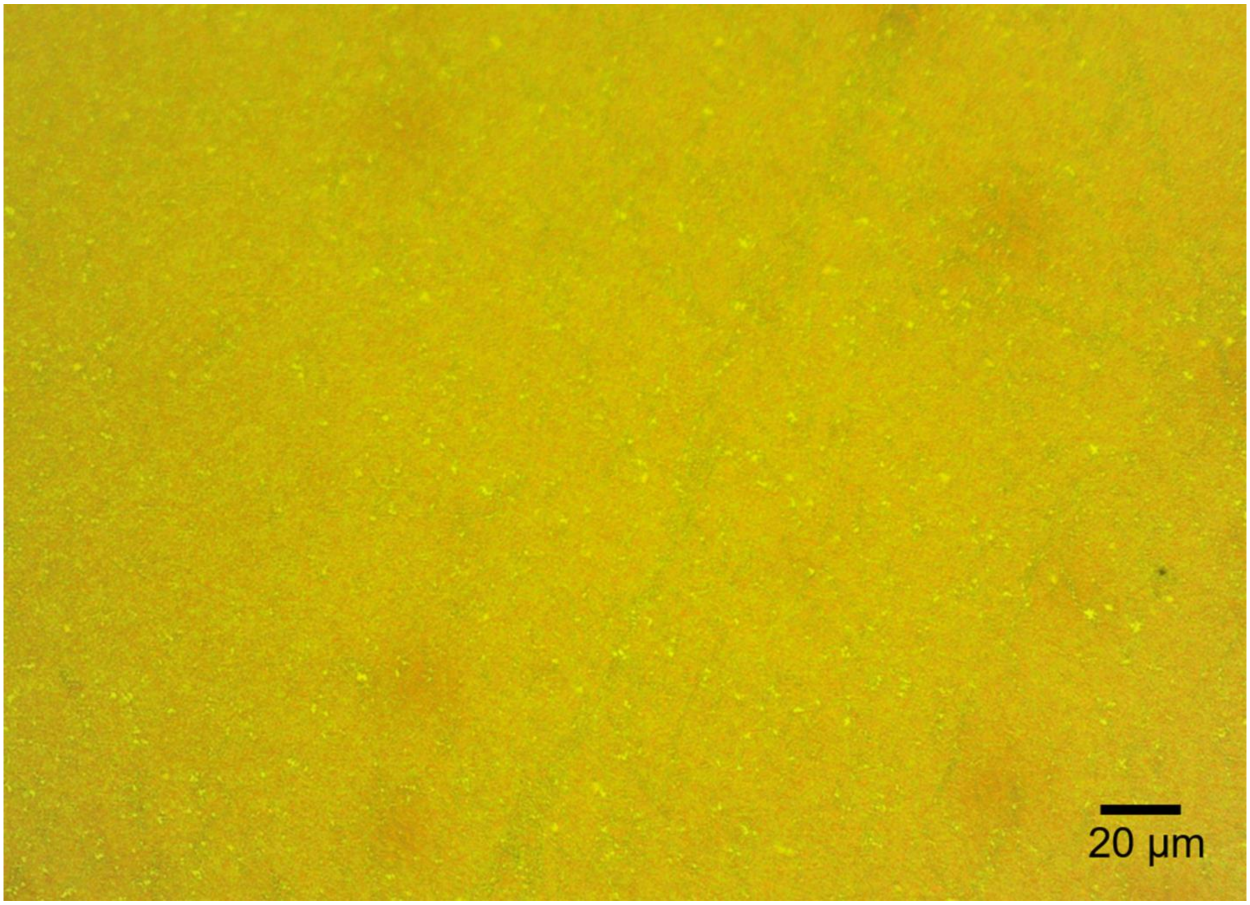

Figure S3. Optical microscope image of the substrate after sEV solution introduction

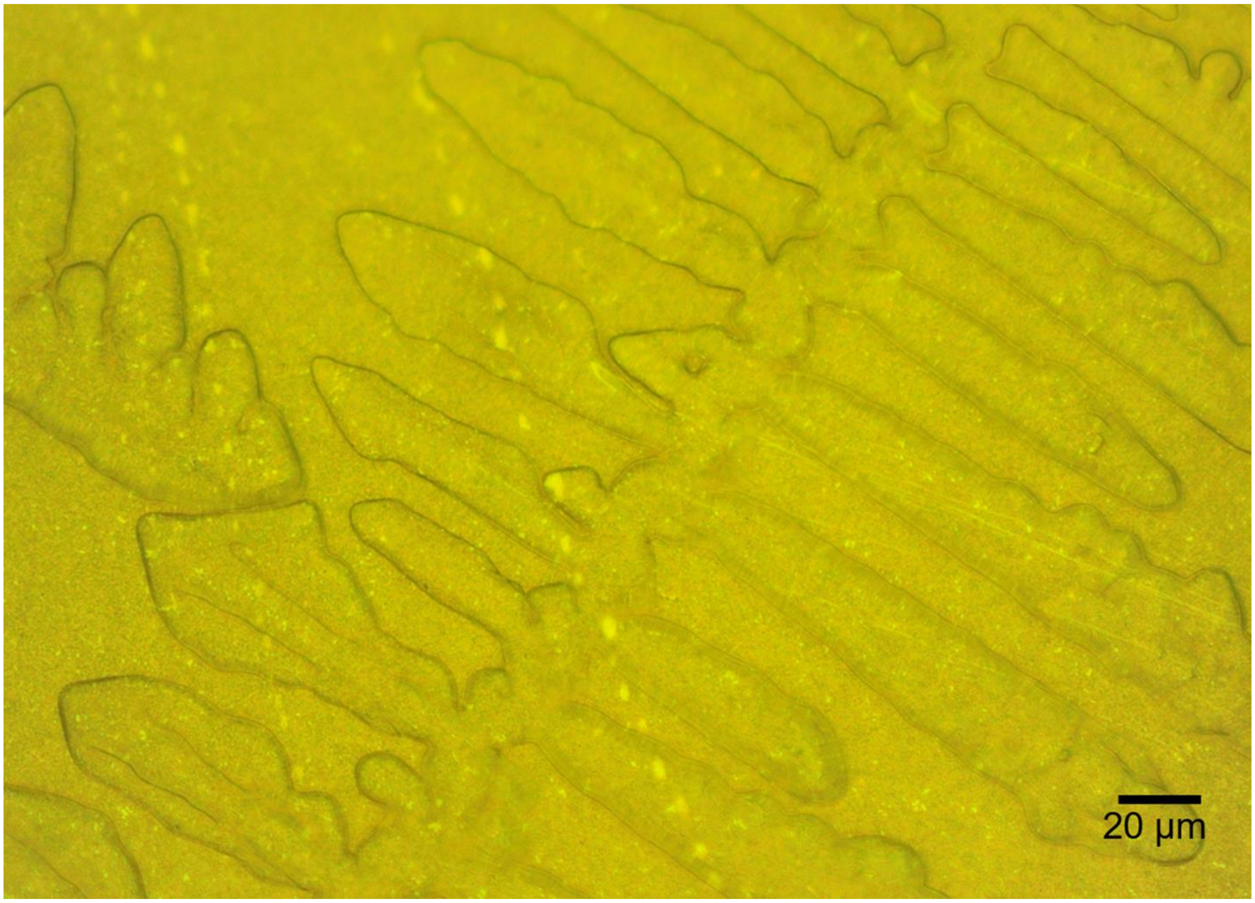

Figure S4. SERS spectra of sEVs common to CRL1739 & CRL5822

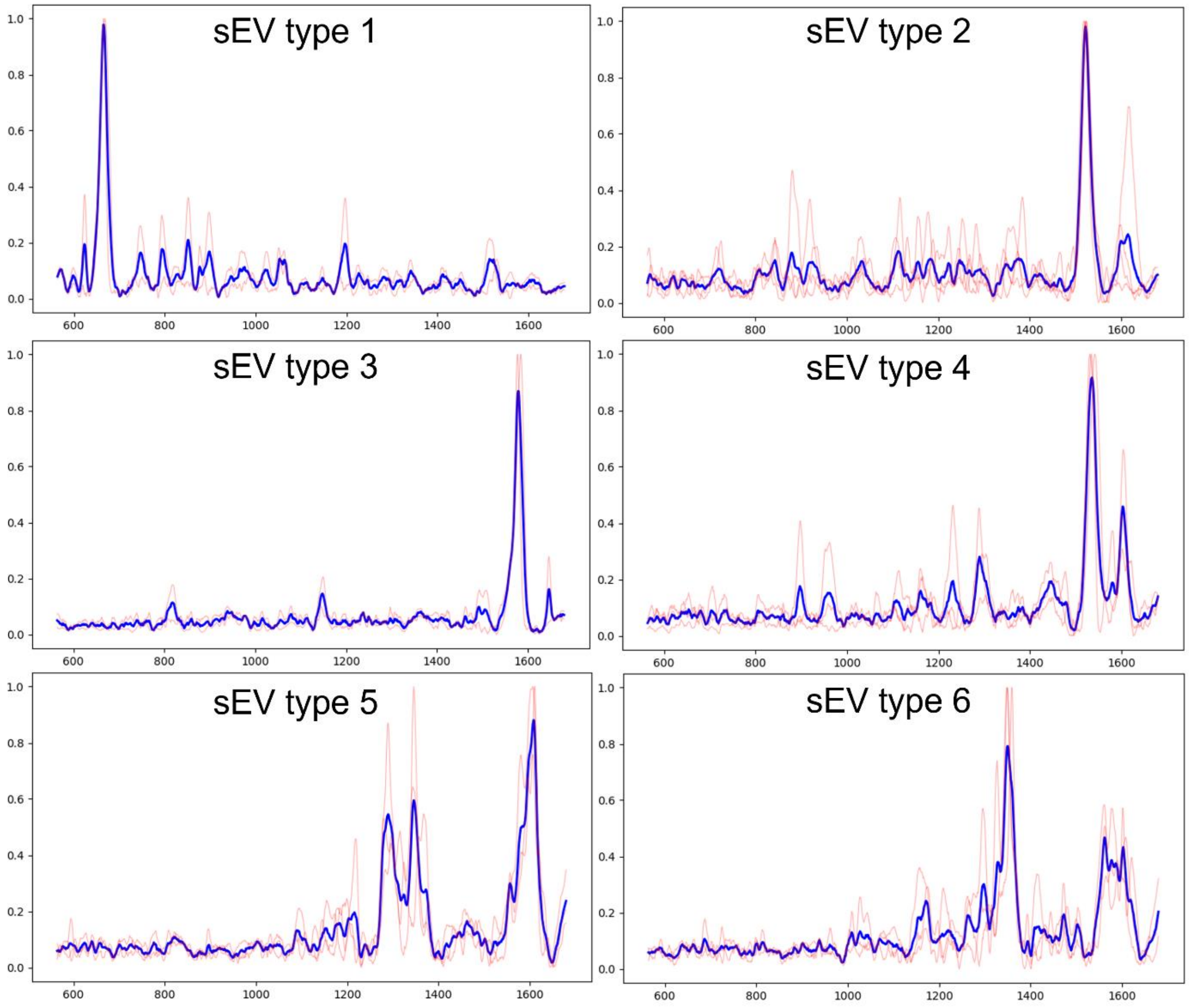

Figure S5. SERS spectra of sEVs common to CRL1739 & CRL7869

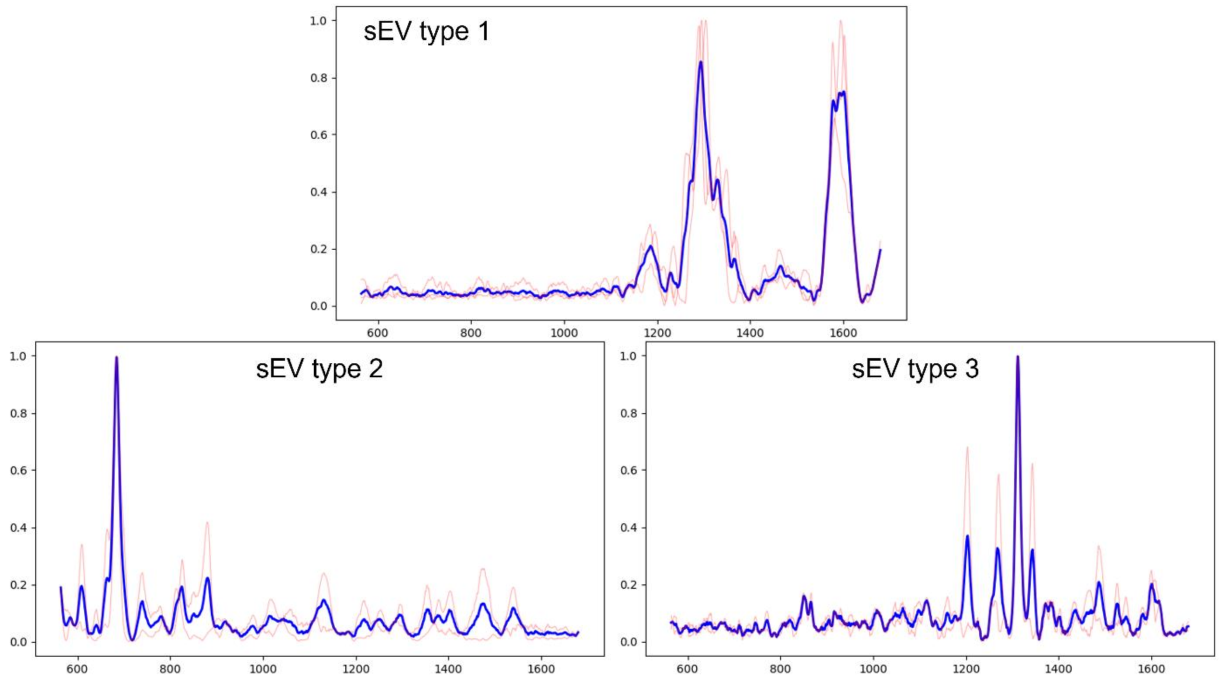

Figure S6. SERS spectra of sEVs common to CRL5822 & CRL7869

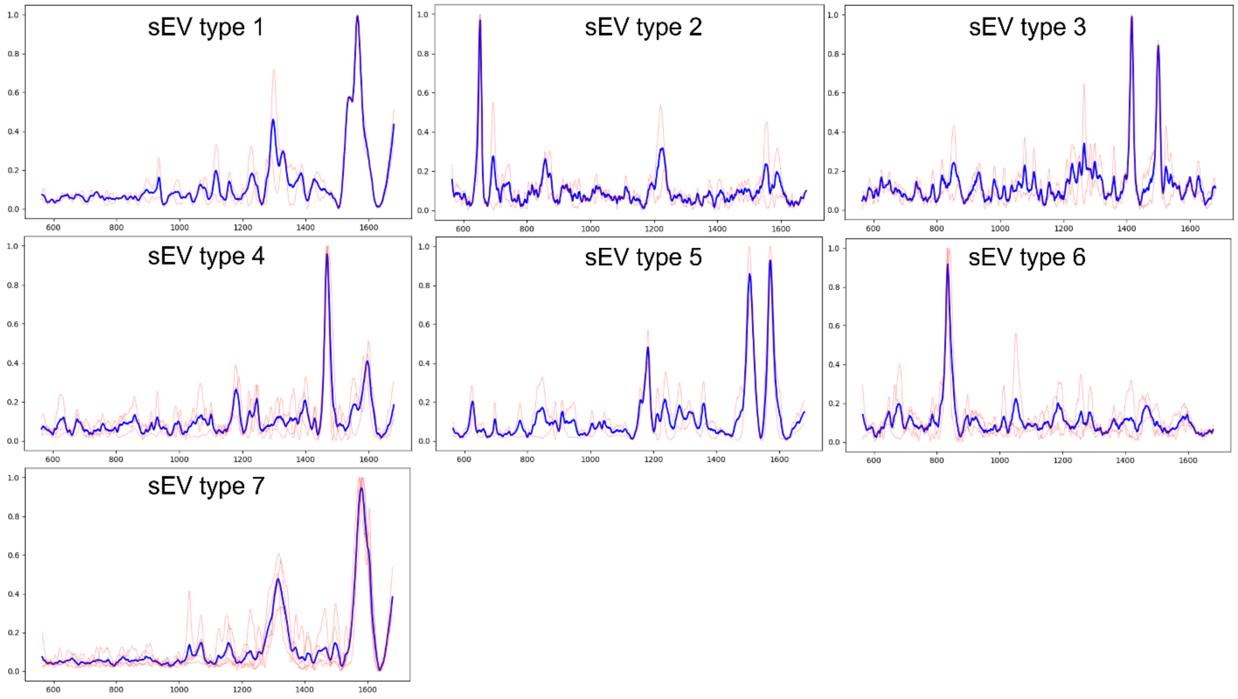

Figure S7. SERS spectra of sEVs common to CRL1739, CRL5822 & CRL7869

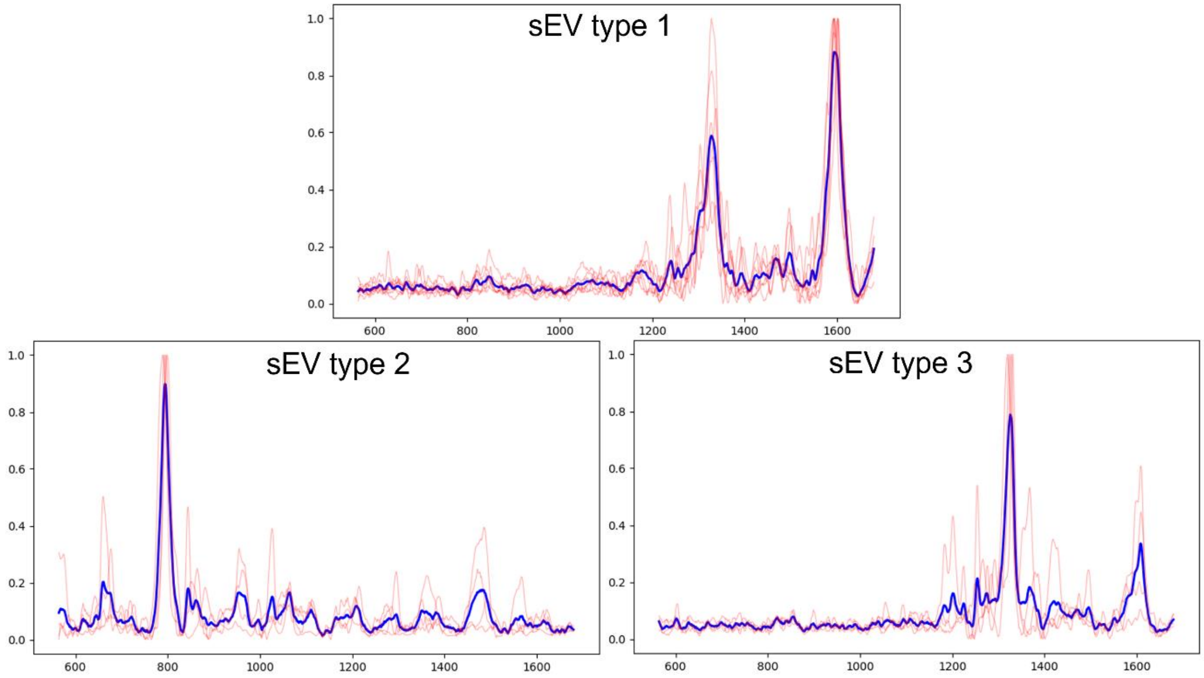

Figure S8. Schematics of the conventional data labeling and data relabeling

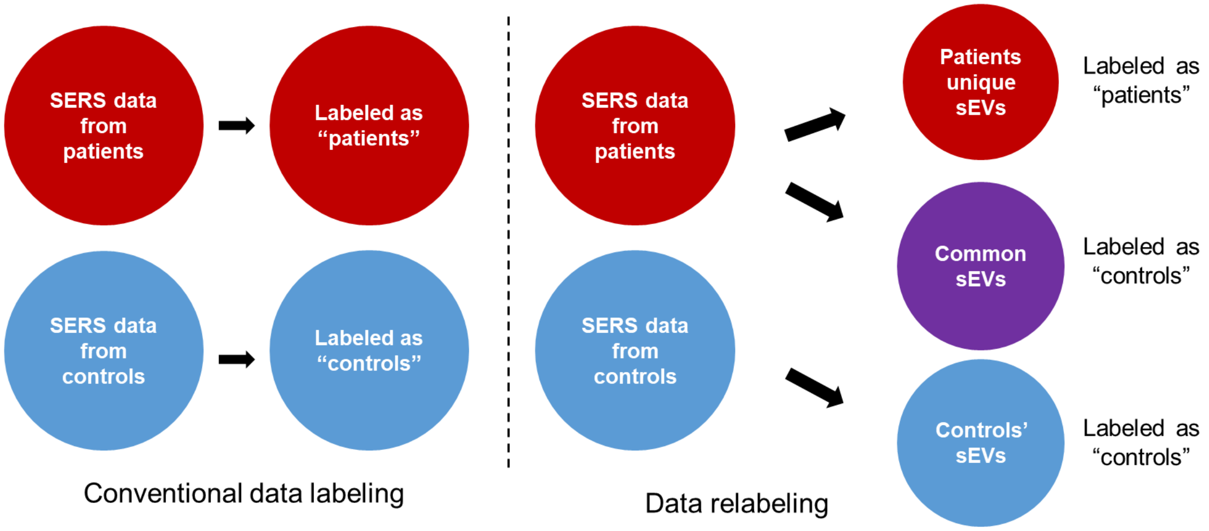

Table S1. Tentative Raman Peak Assignments of sEV type 1

Raman Shift	Peak Assignment	Reference
576 cm^{-1}	Phosphatidylinositol	1
776 cm^{-1}	Phosphatidylinositol	1
941 cm^{-1}	Skeletal modes (polysaccharides)	2
980 cm^{-1}	C-C stretching β-sheet (proteins)	3
1077 cm^{-1}	phospholipids (lipid assignment); (PO_2^-) nucleic acid	4
1169 cm^{-1}	Tyrosine (collagen type I)	5
1281 cm^{-1}	Collagen; Nucleic acids; phosphates	6, 7
1297 cm^{-1}	Palmitic acid	8
1420 cm^{-1}	CH2 scissoring vibration (lipid band)	9
1484 cm^{-1}	G, A (ring breathing modes in the DNA bases)	10
1548 cm^{-1}	Tryptophan	11
1582 cm^{-1}	Phenylalanine	12

Table S2. Tentative Raman Peak Assignments of sEV type 2

Raman Shift	Peak Assignment	Reference
646 cm^{-1}	C-C twisting mode of tyrosine	5
754 cm^{-1}	Symmetric breathing of tryptophan (protein assignment)	12, 11, 5
802 cm^{-1}	Uracil-based ring breathing mode	13
817 cm^{-1}	C-C stretching (collagen assignment)	14
838 cm^{-1}	Deformative vibrations of amine groups	15
928 cm^{-1}	Amino acids proline and valine (protein band)	16
968 cm^{-1}	Lipids	6
1000 cm^{-1}	Phenylalanine	17
1078 cm^{-1}	Phospholipids (lipid assignment)	12
1104 cm^{-1}	Phenylalanine (proteins)	18
1170 cm^{-1}	C-H in-plane bending mode of tyrosine	11, 22
1230 cm^{-1}	Amide III	6
1287 cm^{-1}	Cytosine	19
1458 cm^{-1}	Deoxyribose; Nucleic acid modes	19
1580 cm^{-1}	C-C stretching; C=C bending mode of phenylalanine	15

Table S3. Tentative Raman Peak Assignments of sEV type 3

Raman Shift	Peak Assignment	Reference
880 cm^{-1}	Tryptophan, δ(ring)	2
898 cm^{-1}	Monosaccharides (β-glucose); Adenine	2, 11
914 cm^{-1}	Ribose vibration; one of the distinct RNA modes	10
968 cm^{-1}	Lipids	6
1000 cm^{-1}	Phenylalanine	17
1074 cm^{-1}	Glucose, triglycerides, C-C (lipid)	8
1149 cm^{-1}	Carbohydrates; Glycogen	6
1166 cm^{-1}	Lipids	6
1257 cm^{-1}	A, T (ring breathing modes of the DNA/RNA bases); amide III	10
1287 cm^{-1}	Cytosine	19
1329 cm^{-1}	Purine bases of nucleic acids	20, 21
1383 cm^{-1}	CH_3 band	8
1446 cm^{-1}	CH_3 bending mode of proteins and lipids	14
1586 cm^{-1}	Phenylalanine	5

Table S4. Tentative Raman Peak Assignments of sEV type 4

Raman Shift	Peak Assignment	Reference
621 cm^{-1}	C-C twisting mode of phenylalanine (proteins)	11
646 cm^{-1}	C-C twisting mode of tyrosine	5
700 cm^{-1}	ν(C-S) trans (amino acid methionine)	2
710 cm^{-1}	ν(C-S) trans (amino acid methionine)	2
814 cm^{-1}	Proline, tyrosine, ν2 PO_2^- stretch of nucleic acids	5
830 cm^{-1}	Tyrosine	2
1000 cm^{-1}	Phenylalanine	17
1061 cm^{-1}	C-C in-plane bending; Palmitic acid	23
1123 cm^{-1}	Proteins (protein assignment)	12
1174 cm^{-1}	Tyrosine; Phenylalanine; Cytosine; Guanine	19
1287 cm^{-1}	Proteins, including collagen I	24
1462 cm^{-1}	δCH2, disaccharides, sucrose	2
1550 cm^{-1}	Tryptophan	12

Table S5. Tentative Raman Peak Assignments of sEV type 5

Raman Shift	Peak Assignment	Reference
720 cm^{-1}	DNA; Nucleic acids	24
814 cm^{-1}	Proline, tyrosine, ν2 PO_2^- stretch of nucleic	5
937 cm^{-1}	Collagen; Glycogen; Proline	5
1021 cm^{-1}	Glycogen	6
1058 cm^{-1}	PO_2^- stretching (DNA/RNA)	3
1131 cm^{-1}	Palmitic acid; Fatty acid	1
1204 cm^{-1}	Amide III; Tyrosine; Phenylalanine	7
1313 cm^{-1}	Collagen; Lipid	11
1370 cm^{-1}	T, A, G (ring breathing modes of the DNA/RNA bases)	10
1550 cm^{-1}	Tryptophan	12

Table S6. Tentative Raman Peak Assignments of sEV type 6

Raman Shift	Peak Assignment	Reference
627 cm^{-1}	Glycerol; ν(C-S) gauche (amino acid methionine)	1, 2
741 cm^{-1}	DNA, tryptophan	8
1067 cm^{-1}	Proline (collagen assignment)	14
1177 cm^{-1}	Cytosine, guanine	19
1334 cm^{-1}	Collagen (protein assignment); Nucleic acid	12
1373 cm^{-1}	T, A, G (ring breathing modes of the DNA/RNA bases)	10
1443 cm^{-1}	CH2 deformation (lipids and proteins); Triglycerides (fatty acids)	22, 25
1499 cm^{-1}	C-C stretching	15
1573 cm^{-1}	Guanine, adenine, TRP (protein)	22
1593 cm^{-1}	C=N and C-C stretching	15

Table S7. Tentaive Raman Peak Assignments of sEV type 7

Raman Shift	Peak Assignment	Reference
621 cm^{-1}	C-C twisting mode of phenylalanine (proteins)	11
832 cm^{-1}	Tyrosine	10
898 cm^{-1}	Monosaccharides (β-glucose); Disaccharide (maltose)	2
1000 cm^{-1}	Phenylalanine	17
1078 cm^{-1}	Phospholipids (lipid assignment)	12
1154 cm^{-1}	C-C (and C-N) stretching of proteins	11
1174 cm^{-1}	Tyrosine, phenylalanine, C-H bend (protein)	10
1220 cm^{-1}	C=N=C stretching	15
1297 cm^{-1}	Palmitic acid	8
1501 cm^{-1}	C-C stretching	15
1600 cm^{-1}	Amide I band of proteins	10

Table S8. Tentative Raman Peak Assignments of sEV type 8

Raman Shift	Peak Assignment	Reference
678 cm^{-1}	Ring breathing modes in the DNA bases	10
823 cm^{-1}	Tyrosine (protein assignment)	12
886 cm^{-1}	Disaccharide (cellobiose)	2
1006 cm^{-1}	Phenylalanine, δ(ring)	2
1070 cm^{-1}	Triglycerides (fatty acids)	25
1170 cm^{-1}	C-H in-plane bending mode of tyrosine	11
1200 cm^{-1}	Nucleic acids	7
1220 cm^{-1}	Amide III; T, A (DNA/RNA)	3
1480 cm^{-1}	Amide II	6
1560 cm^{-1}	Tryptophan	26

Table S9. Tentative Raman Peak Assignments of sEV type 9

Raman Shift	Peak Assignment	Reference
646 cm^{-1}	C-C twisting mode of tyrosine	5
813 cm^{-1}	One of the two most distinct peaks for RNA	10
1000 cm^{-1}	Phenylalanine	17
1075 cm^{-1}	C-C (lipid)	17
1153 cm^{-1}	Carbohydrates	6
1184 cm^{-1}	Cytosine, guanine, adenine	22
1257 cm^{-1}	A, T (DNA/RNA bases); Amide III (protein)	10
1298 cm^{-1}	Palmitic acid	3
1465 cm^{-1}	Lipids	6
1481 cm^{-1}	Amide II	6
1540 cm^{-1}	Amide carbonyl group vibrations	27
1578 cm^{-1}	Guanine, adenine	3

Reference

1. Krafft, C., Neudert, L., Simat, T., and Salzer, R. (2005) Near infrared Raman spectra of human brain lipids. *Spectrochim. Acta Mol. Biomol. Spectros.*, 61: 1529–1535.

2. Shetty, G., Kendall, C., Shepherd, N., Stone, N., and Barr, H. (2006) Raman spectroscopy: Elucidation of biochemical changes in carcinogenesis of oesophagus. *Br. J. Canc.*, 94: 1460–1464.

3. Liu, Z., Davis, C., Cai, W., He, L., Chen, X., and Dai, H. (2008) Circulation and long-term fate of functionalized, biocompatible single-walled carbon nanotubes in mice probed by Raman spectroscopy. *Proc. Natl. Acad. Sci. Unit. States Am.*, 105 (5): 1410–1415.

4. Bhattacharjee, T., Kumar, P.,Maru, G., Ingle, A., and Krishna, C.M. (2014) Swiss bare mice: A suitable model for transcutaneous in vivo Raman spectroscopic studies of breast cancer. *Laser. Med. Sci.*, 29 (1): 325–333.

5. Cheng, W.T., Liu, M.T., Liu, H.N., and Lin, S.Y. (2005) Micro-Raman spectroscopy used to identify and grade human skin pilomatrixoma. *Microsc. Res. Tech.*, 68: 75–79.

6. Dukor, R.K. (2002) Vibrational spectroscopy in the detection of cancer. *Biomedical Applications*, 5: 3335–3359.

7. Schulz, H. andBaranska, M. (2007) Identification and quantification of valuable plant substances by IR and Raman spectroscopy. *Vib. Spectros.*, 43: 13–25.

8. Huang, N.Y., Short, M., Zhao, J.H., Wang, H.Q., Lui, H., Korbelik, M., and Zeng, H.S. (2011) Full range characterization of the Raman spectra of organs in a murine model. Optic. Express, 19 (23): 22892–22909.

9. Gniadecka, M., Wulf, H.C., Mortensen, N.N., Nielsen, O.F., and Christensen, D.H. (1997) Diagnosis of basal cell carcinoma by Raman spectroscopy. *J. Raman Spectros.*, 28: 125–129.

10. Chan, J.W., Taylor, D.S., Zwerdling, T., Lane, S.M., Ihara, K., and Huser, T. (2006) Micro-Raman spectroscopy detects individual neoplastic and normal hematopoietic cells. Biophys. J., 90: 648–656.

11. Stone, N., Kendall, C., Shepherd, N., Crow, P., and Barr, H. (2002) Near-infrared Raman spectroscopy for the classification of epithelial pre-cancers and cancers. *J. Raman Spectros.*, 33: 564–573.

12. Huang, Z.W., McWilliams, A., Lui, H., McLean, D.I., Lam, S., and Zeng, H.S. (2003) Nearinfrared Raman spectroscopy for optical diagnosis of lung cancer. *Int. J. Canc.*, 107 (6): 1047–1052.

13. Kendall, C., Day, J., Hutchings, J., Smith, B., Shepherd, N., Barr, H., and Stone, N. (2010)

Evaluation of Raman probe for oesophageal cancer diagnostics. *Analyst*, 135 (12):3038–3041.

14. Bonnier, F. and Byrne, H.J. (2012) Understanding the molecular information contained in principal component analysis of vibrational spectra of biological systems. *Analyst*, 137: 2.

15. Shapiro, A., Gofrit, O.N., Pizov, G., Cohen, J.K., and Maier, J. (2011) Raman molecular imaging: A novel spectroscopic technique for diagnosis of bladder cancer in urine specimens. *Eur. Urol.*, 59 (1): 106–112.

16. Larraona-Puy, M., Ghita, A., Zoladek, A., Perkins, W., Varma, S., Leach, I.H., Koloydenko, A.A., Williams, H., and Notingher, I. (2009) Development of Raman microspectroscopy for automated detection and imaging of basal cell carcinoma. *J. Biomed. Optic.*, 14: 5.

17. Malini, R., Venkatakrishna, K., Kurien, J., Pai, K.M., Rao, L., Kartha, V.B., and Krishna, C.M. (2006) Discrimination of normal, inflammatory, premalignant, and malignant oral tissue: A Raman spectroscopy study. *Biopolymers*, 81 (3): 179–193.

18. Lakshmi, R.J., Kartha, V.B., Krishna, C.M., Solomon, J.G.R., Ullas, G., and Devi, P.U. (2002) Tissue Raman spectroscopy for the study of radiation damage: Brain irradiation ofmice. *Radiat. Res.*, 157 (2): 175–182.

19. Ruiz-Chica, A.J.,Medina, M.A., Sanchez-Jimenez, F., and Ramirez, F.J. (2004) Characterization by Raman spectroscopy of conformational changes on guanine–cytosine and adenine–thymine oligonucleotides induced by aminooxy analogues of spermidine. *J. Raman Spectros.*, 35: 93–100.

20. Wang, H., Huang, N., Zhao, J., Lui, H., Korbelik, M., and Zeng, H. (2011) Depth-resolved in vivo micro-Raman spectroscopy of a murine skin tumor model reveals cancer-specific spectral biomarkers. *J. Raman Spectros.*, 42 (2): 160–166.

21. Viehoever, A.R., Anderson, D., Jansen, D., and Mahadevan-Jansen, A. (2003) Organotypic raft cultures as an effective in vitro tool for understanding Raman spectral analysis of tissue. *Photochem. Photobiol.*, 78: 517–524.

22. Stone, N., Kendall, C., Smith, J., Crow, P., and Barr, H. (2004) Raman spectroscopy for identification of epithelial cancers. *Faraday Discuss.*, 126: 141–157.

23. O Faolain, E., Hunter, M.B., Byrne, J.M., Kelehan, P., McNamara, M., Byrne, H.J., and Lyng, F.M. (2005) A study examining the effects of tissue processing on human tissue sections using vibrational spectroscopy. *Vib. Spectros.*, 38: 121–127.

24. Binoy, J., Abraham, J.P., Joe, I.H., Jayakumar, V.S., Pettit, G.R., and Nielsen, O.F. (2004) NIR-FT Raman and FT-IR spectral studies and ab initio calculations of the anti-cancer drug combretastatin-A4. *J. Raman Spectros.*, 35: 939–946.

25. Silveira, L., Sathaiah, S., Zangaro, R.A., Pacheco, M.T.T., Chavantes, M.C., and Pasqualucci,

C.A.G. (2002) Correlation between near-infrared Raman spectroscopy and the histopathological analysis of atherosclerosis in human coronary arteries. *Laser. Surg. Med.*, 30: 290–297.

26. Sigurdsson, S., Philipsen, P.A., Hansen, L.K., Larsen, J., Gniadecka, M., andWulf, H.C. (2004) Detection of skin cancer by classification of Raman spectra. *IEEE Trans. Biomed. Eng.*, 51: 10.

27. Katainen, E., Elomaa, M., Laakkonen, U.-M., Sippola, E., Niemela, P., Suhonen, J., and Jarvinen, K. (2007) Quantification of the amphetamine content in seized street samples by Raman spectroscopy. *J. Forensic Sci.*, 52 (1): 88–92.
